# Never Miss Neuroleptic Malignant Syndrome! – A Trainee-Led Collaborative Learning and Improvement Programme Initiative

**DOI:** 10.1192/bjo.2026.11274

**Published:** 2026-06-30

**Authors:** Jun Hao Teo, Tze En Tan, Wan Ting Chan, Arvind Rajagopalan, Bharathi Balasundaram

**Affiliations:** 1 Institute of Mental Health, Singapore, Singapore; 2 Changi General Hospital, Singapore, Singapore

## Abstract

**Aims:**

The Collaborative Learning and Improvement Programme (CLIP) is a hospital-wide educational initiative on medication safety, co-visioned and co-produced by interdisciplinary members of the Changi General Hospital (CGH) Medication Safety Committee (MSC). CGH is a public hospital in Singapore, where CLIP has been operational since October 2023 and various educational materials have been implemented into hospital-wide junior, senior doctor and interprofessional education and training. In September 2024, inspired by a departmental case conference which triggered discussions on Neuroleptic Malignant Syndrome (NMS), the MSC Chairperson from the department of Psychological Medicine worked with departmental trainees on developing CLIP educational material on NMS. We aimed to design a CLIP educational infographic on NMS and implement this in hospital-wide mental health training.

**Methods:**

Digital technology-enhanced learning using the infographic, with microlearning as the pedagogy, formed the framework to design the CLIP interprofessional educational infographic.

The design phase involved engaging stakeholders and end-users from the department, including Psychiatry trainees, from September–October 2025. Pharmacist, nursing and medical members of the MSC were engaged at the committee meeting in November 2025 to ensure interdisciplinary stakeholder contribution to designing the infographic. The final version of the infographic was implemented and shared in a mental health training session for the hospital’s Internal Medicine department conducted by a Psychiatry consultant in December 2025.

Qualitative feedback was captured at the planning, design and iteration phases.Qualitative and quantitative methodology was employed to capture end-user feedback from medical doctors in the implementation phase.

**Results:**

Qualitative feedback in the design phase led to various iterations of the infographic content, design and aesthetics, with the finalised infographic incorporating the themes captured from this feedback.In the implementation phase, quantitative feedback captured from end-users showed improvements in self-rated awareness of risk factors (69.6% to 100%) and signs and symptoms (60.9% to 100%) of NMS, and confidence in recognizing and managing NMS (4.3% to 70.6%). All participants rated the infographic as useful in aiding recognition and management of NMS. Qualitative feedback centred on the usefulness of the infographic’s design, organization and informativeness.

**Conclusion:**

We designed a trainee-led infographic on NMS underpinned by interdisciplinary feedback, the implementation of which in the mental health training of general medical doctors has shown promising results, reinforcing the value of the CLIP. The CLIP NMS infographic will be made accessible to all hospital staff via the hospital intranet as a systems intervention and will be incorporated into interdisciplinary CLIP educational programmes.

